# Supplementary material for: The pooled prevalence of attention-deficit/hyperactivity disorder among children and adolescents in Ethiopia: A systematic review and meta-analysis
Source: PLoS One. 2024 Jul 18;19(7):e0307173. doi: 10.1371/journal.pone.0307173 (PMC11257254; doi:10.1371/journal.pone.0307173)
Supplement: S1 Checklist — (DOCX) [file pone.0307173.s001.docx]

| **Section and Topic** | **Item #** | **Checklist item** | **Location where item is reported** |
| --- | --- | --- | --- |
| **TITLE: The pooled prevalence of attention-deficit/hyperactivity disorder among children and adolescents in Ethiopia: A Systematic Review and Meta-Analysis.** | | |  |
| Title | 1 | Identify the report as a systematic review. | Yes: Page 1: line 1 |
| **ABSTRACT** | | |  |
| Abstract | 2 | See the PRISMA 2020 for Abstracts checklist. | From pages 2-3, from lines 21-45 |
| **INTRODUCTION** | | |  |
| Rationale | 3 | Describe the rationale for the review in the context of existing knowledge. | Pages 5: from lines 91-99 |
| Objectives | 4 | Provide an explicit statement of the objective(s) or question(s) the review addresses. | Pages 5: from lines 91-99 |
| **METHODS** | | |  |
| Eligibility criteria | 5 | Specify the inclusion and exclusion criteria for the review and how studies were grouped for the syntheses. | Pages 6: from lines 110-116 |
| Information sources | 6 | Specify all databases, registers, websites, organisations, reference lists and other sources searched or consulted to identify studies. Specify the date when each source was last searched or consulted. | Pages 5 : from lines 101-102 |
| Search strategy | 7 | Present the full search strategies for all databases, registers and websites, including any filters and limits used. | Pages 5 : from lines 101-110 and indicated in additional table 1 |
| Selection process | 8 | Specify the methods used to decide whether a study met the inclusion criteria of the review, including how many reviewers screened each record and each report retrieved, whether they worked independently, and if applicable, details of automation tools used in the process. | Pages 7 : from lines 130-135 |
| Data collection process | 9 | Specify the methods used to collect data from reports, including how many reviewers collected data from each report, whether they worked independently, any processes for obtaining or confirming data from study investigators, and if applicable, details of automation tools used in the process. | Pages 6: from lines 117-121 |
| Data items | 10a | List and define all outcomes for which data were sought. Specify whether all results that were compatible with each outcome domain in each study were sought (e.g. for all measures, time points, analyses), and if not, the methods used to decide which results to collect. | Pages 7 : from lines 130-135 |
|  | 10b | List and define all other variables for which data were sought (e.g. participant and intervention characteristics, funding sources). Describe any assumptions made about any missing or unclear information. | NA |
| Study risk of bias assessment | 11 | Specify the methods used to assess risk of bias in the included studies, including details of the tool(s) used, how many reviewers assessed each study and whether they worked independently, and if applicable, details of automation tools used in the process. | Pages 8 from lines 153-155 |
| Effect measures | 12 | Specify for each outcome the effect measure(s) (e.g. risk ratio, mean difference) used in the synthesis or presentation of results. | Pages 7 : from lines 130-135 |
| Synthesis methods | 13a | Describe the processes used to decide which studies were eligible for each synthesis (e.g. tabulating the study intervention characteristics and comparing against the planned groups for each synthesis (item #5)). | Yes , pages 7, lines 137-149 |
|  | 13b | Describe any methods required to prepare the data for presentation or synthesis, such as handling of missing summary statistics, or data conversions. | Trim and fill analysis |
|  | 13c | Describe any methods used to tabulate or visually display results of individual studies and syntheses. | Pages 7,from lines Yes , pages 7, lines 146 |
|  | 13d | Describe any methods used to synthesize results and provide a rationale for the choice(s). If meta-analysis was performed, describe the model(s), method(s) to identify the presence and extent of statistical heterogeneity, and software package(s) used. | Models: Pages 7, from lines 147-149  heterogeneity, and software package: pages 7, from lines 139- 140 |
|  | 13e | Describe any methods used to explore possible causes of heterogeneity among study results (e.g. subgroup analysis, meta-regression). | Pages 7, from lines 140-141 |
|  | 13f | Describe any sensitivity analyses conducted to assess robustness of the synthesized results. | Pages 7, from lines 142-143 |
| Reporting bias assessment | 14 | Describe any methods used to assess risk of bias due to missing results in a synthesis (arising from reporting biases). | Pages 7, from line 144-145 |
| Certainty assessment | 15 | Describe any methods used to assess certainty (or confidence) in the body of evidence for an outcome. | Pages 7, from line 145-146 |
| **RESULTS** | | |  |
| Study selection | 16a | Describe the results of the search and selection process, from the number of records identified in the search to the number of studies included in the review, ideally using a flow diagram. | Indicated by **Fig 1** |
|  | 16b | Cite studies that might appear to meet the inclusion criteria, but which were excluded, and explain why they were excluded. | Pages 8, from lines 151-163 and it Indicated by **Fig 1** |
| Study characteristics | 17 | Cite each included study and present its characteristics. | Pages 8, from lines161 and it was Indicated in **Table 1** |
| Risk of bias in studies | 18 | Present assessments of risk of bias for each included study. | Pages 10: from lines 188-192 |
| Results of individual studies | 19 | For all outcomes, present, for each study: (a) summary statistics for each group (where appropriate) and (b) an effect estimate and its precision (e.g. confidence/credible interval), ideally using structured tables or plots. | Indicated in Forest plot: in fig: 2 ,and **table 4** |
| Results of syntheses | 20a | For each synthesis, briefly summarise the characteristics and risk of bias among contributing studies. | Pages 10, indicated under **fig 4** funnel plot was presented and trim and fill analysis was presented |
|  | 20b | Present results of all statistical syntheses conducted. If meta-analysis was done, present for each the summary estimate and its precision (e.g. confidence/credible interval) and measures of statistical heterogeneity. If comparing groups, describe the direction of the effect. | - The pooled prevalence of attention-deficit/hyperactivity disorder among children and adolescents in Ethiopia: A Systematic Review and Meta-Analysis: Pages 9, from lines 168- 170 and indicated in **Fig ;2**  -the predictors : from pages 9 from lines 192-205 and indicated **in table 4**  -sub group analysis- pages 9, from lines 172-174 and indicated in **table 2** |
|  | 20c | Present results of all investigations of possible causes of heterogeneity among study results. | pages 9, from lines 179-182 and indicated **in table 3** |
|  | 20d | Present results of all sensitivity analyses conducted to assess the robustness of the synthesized results. | pages 9, from lines 174-177 and presented in **fig 3** |
| Reporting biases | 21 | Present assessments of risk of bias due to missing results (arising from reporting biases) for each synthesis assessed. | Page 10- f bias was corrected using trim and fill analysis |
| Certainty of evidence | 22 | Present assessments of certainty (or confidence) in the body of evidence for each outcome assessed. | Yes : the effect sizes were presented with its 95%CI |
| **DISCUSSION** | | |  |
| Discussion | 23a | Provide a general interpretation of the results in the context of other evidence. | Pages 12-13 : from lines 213- 251 |
|  | 23b | Discuss any limitations of the evidence included in the review. | Pages 14: from lines 256-261 |
|  | 23c | Discuss any limitations of the review processes used. | Pages 14: from lines 256-261 |
|  | 23d | Discuss implications of the results for practice, policy, and future research. | Pages :13 : from lines 251-254 |
| **OTHER INFORMATION** | | |  |
| Registration and protocol | 24a | Provide registration information for the review, including register name and registration number, or state that the review was not registered. | Pages 15:lines 291 |
|  | 24b | Indicate where the review protocol can be accessed, or state that a protocol was not prepared. | Pages 15:lines 291 |
|  | 24c | Describe and explain any amendments to information provided at registration or in the protocol. | NA |
| Support | 25 | Describe sources of financial or non-financial support for the review, and the role of the funders or sponsors in the review. | Pages 15:lines 290 |
| Competing interests | 26 | Declare any competing interests of review authors. | Pages 15:lines 287 |
| Availability of data, code and other materials | 27 | Report which of the following are publicly available and where they can be found: template data collection forms; data extracted from included studies; data used for all analyses; analytic code; any other materials used in the review. | Pages 15:lines 287 |

*From:*  Page MJ, McKenzie JE, Bossuyt PM, Boutron I, Hoffmann TC, Mulrow CD, et al. The PRISMA 2020 statement: an updated guideline for reporting systematic reviews. BMJ 2021;372:n71. doi: 10.1136/bmj.n71

For more information, visit: <http://www.prisma-statement.org/>
