# Supplementary material for: The pooled prevalence of attention-deficit/hyperactivity disorder among children and adolescents in Ethiopia: A systematic review and meta-analysis
Source: PLoS One. 2024 Jul 18;19(7):e0307173. doi: 10.1371/journal.pone.0307173 (PMC11257254; doi:10.1371/journal.pone.0307173)
Supplement: S1 Table — (DOCX) [file pone.0307173.s002.docx]

S1 Table: Search terms summary for the pooled prevalence of attention-deficit/hyperactivity among children and adolescents, Ethiopia, 2024.

| **Database** |  | **Query** | **Items found** |
| --- | --- | --- | --- |
| PubMed | **#1** | **(((Prevalence) OR (magnitude)) OR (incidence)) OR (Epidemiology)** | 622,822 |
|  | **#2** | **((((Attention Deficit Hyperactivity Disorder) OR (ADHD)) OR (ADDH)) OR (behavioral Disorder)) OR (neurodevelopmental disorder)** | 1,580,255 |
|  | **#3** | **(((children) OR (pediatrics)) OR (under five-children)) OR (Adolescents)** | 4,760,423 |
|  | #4 | **Ethiopia** | 42,309 |
|  | **#5** | **#1 AND #2 AND #3 AND #4** | **441** |
| HINARI |  | ((Prevalence) OR (magnitude) OR (Epidemiology)) AND ((Attention Deficit Hyperactivity Disorder) OR (ADHD) OR (neurodevelopmental disorder) OR (behavioral Disorder)) AND (children) AND (Adolescents) AND (Ethiopia) | **42 results** |
| Science Direct |  | (" Prevalence ") AND ( " Attention Deficit Hyperactivity Disorder " OR " neurodevelopmental disorder" ) AND ("Children" OR "Adolescent") AND ("Ethiopia") | **189** |
| Psych INFO |  | Prevalence OR Any Field: magnitude OR Any Field: Epidemiology AND Any Field: Attention Deficit Hyperactivity Disorder OR Any Field: ADHD OR Any Field: behavioral Disorder AND Any Field: children OR Any Field: Adolescents AND Any Field: Ethiopia | **823** |
| Google Scholar and cross reference |  | Prevalence OR magnitude OR Epidemiology AND Attention Deficit Hyperactivity Disorder OR ADHD OR neurodevelopmental disorder OR behavioral Disorder AND children AND Adolescents AND Ethiopia. | **107** |
| African journal online |  | Prevalence OR magnitude OR Epidemiology AND Attention Deficit Hyperactivity Disorder OR ADHD OR neurodevelopmental disorder OR behavioral Disorder AND children AND Adolescents AND Ethiopia. | **10** |
| **Total** | | | **1,612** |
