# Supplementary material for: The pooled prevalence of attention-deficit/hyperactivity disorder among children and adolescents in Ethiopia: A systematic review and meta-analysis
Source: PLoS One. 2024 Jul 18;19(7):e0307173. doi: 10.1371/journal.pone.0307173 (PMC11257254; doi:10.1371/journal.pone.0307173)
Supplement: S2 Table — (DOCX) [file pone.0307173.s003.docx]

**S2 Table:** Critical appraisal of studies included in the systematic review and meta-analysis for pooled prevalence of attention-deficit/hyperactivity disorder among children and adolescents in Ethiopia, 2024.

| Include studies | **Eight JBI Critical Appraisal Checklist for cross-sectional Studies: The tool has Yes, No, Unclear, and Not Applicable options: “1” is given for “Yes” and “0” is given for other options** | | | | | | | | | | | | | | | | |
| --- | --- | --- | --- | --- | --- | --- | --- | --- | --- | --- | --- | --- | --- | --- | --- | --- | --- |
|  | Q1 | | Q2 | | Q3 | | Q4 | | Q5 | | Q6 | | Q7 | | Q8 | | Overall appraisal |
|  | R1 | R2 | R1 | R2 | R1 | R2 | R1 | R2 | R1 | R2 | R1 | R2 | R1 | R2 | R1 | R2 |  |
| Lola, et al (2019) | Y | Y | Y | Y | Y | Y | Y | Y | Y | Y | Y | Y | Y | Y | Y | Y | 8(100%) |
| Benti , et al (2021) | Y | Y | N | U | Y | Y | Y | Y | Y | Y | Y | Y | Y | Y | Y | Y | 7(87.5%) |
| Mulat, et al(2021) | Y | Y | Y | Y | Y | Y | Y | Y | Y | Y | Y | Y | Y | Y | Y | Y | 8(100%) |
| Aliye, et al (2023) | Y | Y | Y | Y | Y | Y | Y | Y | Y | Y | Y | Y | Y | Y | Y | Y | 8(100%) |
| Kassa , et al (2018) | Y | Y | Y | Y | Y | Y | Y | Y | Y | N | N | Y | Y | Y | N | Y | 6.5(81.25%) |
| Tiruneh, et al (2015) | N | N | Y | Y | Y | Y | Y | Y | Y | Y | Y | Y | Y | Y | Y | Y | 7(87.5%) |
| Mulu, et al (2021) | Y | Y | Y | N | Y | Y | Y | Y | Y | Y | Y | Y | Y | Y | Y | Y | 7.5(93.75%) |
