# Supplementary material for: The pooled prevalence of attention-deficit/hyperactivity disorder among children and adolescents in Ethiopia: A systematic review and meta-analysis
Source: PLoS One. 2024 Jul 18;19(7):e0307173. doi: 10.1371/journal.pone.0307173 (PMC11257254; doi:10.1371/journal.pone.0307173)
Supplement: Attachment — Submitted filename: Response to reviewers final.docx [file pone.0307173.s004.docx]

**Author response to Editor and reviewers**

**Dear Editor and Reviewers,**

Thank you very much for your email incorporating the insight of the editor and reviewer’s comments. We, the authors, would like to express our gratitude to you for the insightful and constructive review that has led to the great improvement of our paper entitled **“The Pooled prevalence of attention-deficit/hyperactivity disorder among Children and adolescents in Ethiopia: A Systematic Review and Meta-Analysis.**

**.** “We declared that all the data underlying the results presented in the study are publicly available in Harvard Dataverse Network repository: <https://doi.org/10.7910/DVN/4FYYGN>. Moreover, we didn’t receive any funds to conduct this study. We have carefully reviewed the comments given by the editor, and reviewers and revised the manuscript accordingly. Our responses are given in a point-by-point manner for the editor, and reviewers’ comments using the Author’s response to reviewer form. If you have any concerns to be addressed, we are happy to consider them.

Best regards!

**Academic editor comments and respective author’s response**

**Editor comment 1:** If you would like to make changes to your financial disclosure, please include your updated statement in your cover letter. Guidelines for resubmitting your figure files are available below the reviewer comments at the end of this letter.

**AuthorsResponse:** Thanks very much for this comment. Dear, editors, we didn’t receive any funds to conduct this study. Figure caption was done in the main documents and figures were uploaded in tiff format.

**Comments:** The [PLOS Data policy](http://www.plosone.org/static/policies.action#sharing) requires authors to make all data underlying the findings described in their manuscript fully available without restriction, with rare exception (please refer to the Data Availability Statement in the manuscript PDF file). The data should be provided as part of the manuscript or its supporting information, or deposited to a public repository. For example, in addition to summary statistics, the data points behind means, medians and variance measures should be available. If there are restrictions on publicly sharing data—e.g. participant privacy or use of data from a third party—those must be specified.

**AuthorsResponse:** Thanks very much for this comment. Dear, editors, we didn’t receive any funds to conduct this study. The data used for this study was deposited at Harvard Dataverse Network repository: <https://doi.org/10.7910/DVN/4FYYGN>..

**Reviewer #1 comments and an author response**

Your systematic review and meta-analysis is interesting dealing with gathering evidence on ADHD in Ethiopia

**Authors:** I would like to express my heart-felt thanks for your devoted time to review our manuscript

**Reviewer comment #1:** Into the introduction, some information about ADHD in adulthood are interesting to take into account. For this reason, you could take some information by <https://doi.org/10.3390/educsci13010037>;:

**Authors Response:** dear reviewer, we thank you heartily for your constructive comments. As per your suggestion we incorporated in the revised manuscript, please, look on page 5 from lines numbers 86-89

**Comment #1:** The manuscript demonstrates strong writing and methodological validity, although it's worth noting that the meta-analysis was based on only 7 studies, which may be seen as a limitation. Additionally, the evidence presented suggests no significant variations in ADHD prevalence between the Ethiopian population and global trends, particularly in terms of gender disparities or exacerbating factors. Consequently, the study doesn't significantly contribute to the existing scientific discourse.

To enhance the originality of the study, one suggestion is to incorporate a section that systematically reviews or narratively explores the primary treatment modalities available for Ethiopian children and adolescents with ADHD. This addition could introduce a novel perspective and broaden the scope of the research, potentially offering valuable insights into diverse cultural contexts and treatment approaches.

**Authors Response:** Thanks very much for your comments and concerns. We accepted your comments. Dear, reviewer, we searched as much as our best for studies conducted on treatment modalities of ADHD among children and adolescents in Ethiopia, however, we can’t access any published study or literature on treatment modalities of ADHD in Ethiopia. Such that, we can’t review the primary treatment modalities of ADHD among children and adolescents in Ethiopia. Dear reviewer, in this regard, we considered your comments as a major limitation of our study and we incorporated under our limitation (please look on page from lines numbers 290-293). Moreover, we recommended further studies on the treatment modality of ADHD and its effects among children and adolescents with ADHD (please look, on page 15 from lines numbers 302-303).

Dear reviewer, this indicates that ADHD is negated in Ethiopia. Dear reviewer, in our study, at least, we disclosed the burden of ADHD among children in Ethiopia, which can alarm the governments and urge the policymaker to endorse strategies or programs, and treatment guidelines related to ADHD to improve the quality of children with ADHD and to prevent the Lifespan impact of ADHD. In this regard, this study may have importance in a country, which has limited evidence regarding ADHD.

**Dear reviewer**, having this, we are ready to consider your further recommendation for any reservation do you have.

**Response to Reviewer #2:**

**Reviewer:** thank you for submitting your work to the journal PLOS ONE and for focusing your study on the topic of ADHD, which is increasingly relevant and at the center of scientific debate. The work sheds important light on the prevalence and associated factors of ADHD among children and adolescents in Ethiopia.
Thank you for your contribution to this essential field of research.
However, some of the steps described within this project need further clarification.
In particular, the following observations could go a long way towards clarifying certain aspects that need to be revised and reformulated.

**Authors:** I would like to express my heart-felt thanks for your devoted time to review our manuscript

**Comment 1:** The references section should be formatted according to the PLOS ONE guidelines, please refer to <https://journals.plos.org/plosone/s/submission-guidelines> .

**Authors Response:** Thanks very much, for your concern and comments, dear reviewer, amendment was done based on the submission-guidelines on reference No.1 and 2

2. The denomination and description of the table should be placed below it, according to the guidelines.

**Authors Response:** Thanks very much, for your concern and comments. Dear reviewer, descriptions were incorporated in the revised manuscript for supplemental tables and figures, which are not uploaded as supplemental material or uploaded separately as figures.

**Comment 2:**  Given the lack of information due to various reasons in Ethiopia and the need to demonstrate to policymakers, researchers, program implementers, and other responsible bodies the importance of understanding the pooled prevalence of ADHD among children and adolescents and identifying its associated factors, it is crucial to highlight the future perspectives for these children as they transition into adulthood, in order to remark the impact of this clinical condition. For more detailed insights, please refer to <https://doi.org/10.3390/educsci13010037>.

**Authors Response:** dear reviewer, we thank you heartily for your constructive comments. As per your suggestion, we incorporated it in the revised manuscript, please, look on page 5 from lines numbers 86-89

**Comment 3:**  I would also suggest including within the introductory section the issue regarding parents with psychopathology in a more cohesive manner, as this topic is mentioned only in the abstract and conclusions without being well integrated into the context with specific references (e.g. 46-47; 268-269).

**Authors Response:** dear reviewer, we thank you heartily for your constructive comments. As per your suggestion, we incorporated it in the revised manuscript, please, look on page 4 from lines numbers 73-79
