# Supplementary material for: The pooled prevalence of attention-deficit/hyperactivity disorder among children and adolescents in Ethiopia: A systematic review and meta-analysis
Source: PLoS One. 2024 Jul 18;19(7):e0307173. doi: 10.1371/journal.pone.0307173 (PMC11257254; doi:10.1371/journal.pone.0307173)
Supplement: Attachment — Submitted filename: Response to reviewers final1.docx [file pone.0307173.s005.docx]

**Dear Editor**

Thank you very much for your email incorporating the insight of the editor and reviewer’s comments. We, the authors, would like to express our gratitude to you for the insightful and constructive review that has led to the great improvement of our paper entitled **“The Pooled prevalence of attention-deficit/hyperactivity disorder among Children and adolescents in Ethiopia: A Systematic Review and Meta-Analysis.**

**.** “We declared that all the data underlying the results presented in the study are publicly available in Harvard Dataverse Network repository: <https://doi.org/10.7910/DVN/4FYYGN>. Moreover, we didn’t receive any funds to conduct this study. We have carefully reviewed the comments given by the editor, and reviewers and revised the manuscript accordingly. Our responses are given in a point-by-point manner for the editor, and reviewers’ comments using the Author’s response to reviewer form. If you have any concerns to be addressed, we are happy to consider them.

Best regards!

**Academic editor comments and respective author’s response**

**Editor comment 1:** Please review your reference list to ensure that it is complete and correct. If you have cited papers that have been retracted, please include the rationale for doing so in the manuscript text, or remove these references and replace them with relevant current references. Any changes to the reference list should be mentioned in the rebuttal letter that accompanies your revised manuscript. If you need to cite a retracted article, indicate the article’s retracted status in the References list and also include a citation and full reference for the retraction notice.

**AuthorsResponse:** Thanks very much for this comments. Dear editors, all references were checked and there were no retracted papers or article. Dear editors, please be noted that all references were cited using EndNote software.

**Comments:** The [PLOS Data policy](http://www.plosone.org/static/policies.action#sharing) requires authors to make all data underlying the findings described in their manuscript fully available without restriction, with rare exception (please refer to the Data Availability Statement in the manuscript PDF file). The data should be provided as part of the manuscript or its supporting information, or deposited to a public repository. For example, in addition to summary statistics, the data points behind means, medians and variance measures should be available. If there are restrictions on publicly sharing data—e.g. participant privacy or use of data from a third party—those must be specified.

**AuthorsResponse:** Thanks very much for this comment. Dear, editors, we didn’t receive any funds to conduct this study. The data used for this study was deposited at Harvard Dataverse Network repository: <https://doi.org/10.7910/DVN/4FYYGN>..

**Comments** : While revising your submission, please upload your figure files to the Preflight Analysis and Conversion Engine (PACE) digital diagnostic tool, <https://pacev2.apexcovantage.com/>. PACE helps ensure that figures meet PLOS requirements. To use PACE, you must first register as a user. Registration is free. Then, login and navigate to the UPLOAD tab, where you will find detailed instructions on how to use the tool. If you encounter any issues or have any questions when using PACE, please email PLOS at [figures@plos.org](mailto:figures@plos.org). Please note that Supporting Information files do not need this step.

Authors response : Dear editors ,thank you for your comments, we upload tiff format figures.

**Reviewer #1 comments and an author response**

Reviewer #1: Dear authors,

thank you for the revisions made. However, please review the form of the references, as it may not be correct for the journal. It is suggested to use reference software (e.g. Zotero, Mendeley).

Authors response: Dear reviewer thank you for comments and concern, we used EndNote software which is one of the popular citation manager as like Zotero, Mendeley. We have no experience on Zotero, Mendeley. Dear reviewer, we have article that published in **Plos One** and other journal which was cited using EndNote as like as our current manuscript.

Reviewer #2: Thank you for reviewing your work.

In my opinion, the manuscript aligns with the journal's standards.

Authors response: Dear reviewer thank you very much for your devoted time to review our manuscript.
